# Comparative Evaluation of ChatGPT, Google Translate, and UD Talk for Chinese-to-Japanese Translation in Cardiology and Pulmonology Outpatient Consultations: Prospective Observational Study

**DOI:** 10.2196/93082

**Published:** 2026-06-18

**Authors:** Ke-Yun Chao, Chia-Hui Huang, Wei-Lun Liu, Chao-Yu Chen, Ai Yin Lim, Yi-Zhen Chu, Atsuko Sasaoka, Chung-Yu Lin

**Affiliations:** 1 Department of Respiratory Therapy College of Medicine Fu Jen Catholic University New Taipei City Taiwan; 2 Telehealth Center Fu Jen Catholic University Hospital, Fu Jen Catholic University New Taipei City Taiwan; 3 Cardiovascular and Pulmonary Rehabilitation Center Fu Jen Catholic University Hospital, Fu Jen Catholic University New Taipei City Taiwan; 4 Center for Health Research and Innovation Fu Jen Catholic University New Taipei City Taiwan; 5 Graduate Institute of Cross-Cultural Studies Fu Jen Catholic University New Taipei City Taiwan; 6 School of Medicine College of Medicine Fu Jen Catholic University New Taipei City Taiwan; 7 Department of Critical Care Medicine Fu Jen Catholic University Hospital, Fu Jen Catholic University New Taipei City Taiwan; 8 Department of Life Science Fu Jen Catholic University New Taipei City Taiwan; 9 Department of Physical Therapy College of Medical and Health Science Asia University Taichung Taiwan; 10 Division of Cardiology Fu Jen Catholic University Hospital, Fu Jen Catholic University New Taipei City Taiwan; 11 Department of Medical Education Fu Jen Catholic University Hospital, Fu Jen Catholic University New Taipei City Taiwan

**Keywords:** language barrier, medical communication, medical interpretation, cardiopulmonary disease, machine translation, artificial intelligence, AI

## Abstract

**Background:**

Language barriers between health care providers and patients can compromise communication quality, patient safety, and health care equity. When professional interpreter services are limited, particularly in outpatient settings, artificial intelligence–based translation tools may serve as supplementary communication aids.

**Objective:**

This study compared 3 Chinese-to-Japanese translation tools (ChatGPT, Google Translate, and UD Talk) in terms of their performance in real-world cardiology and pulmonology outpatient consultations.

**Methods:**

In this single-center prospective observational study, audio-recorded outpatient consultations between December 2024 and November 2025 were analyzed. Verbatim physician-patient dialogues were translated using the 3 systems. Selected dialogue exchanges were evaluated by professional medical interpreters for translation accuracy and by Japanese-speaking lay participants for translation satisfaction using a 6-point Likert scale. Similarity of translation outputs was assessed at the dialogue exchange level.

**Results:**

A total of 20 outpatient consultations comprising 2450 dialogue exchanges were analyzed. ChatGPT had significantly higher translation accuracy and satisfaction scores than Google Translate and UD Talk, with median scores of 5.0 (IQR 4.0-5.0) for both outcomes compared with 2.0 (IQR 1.0-3.0) for Google Translate and UD Talk (*P*<.001), and maintained a consistently high performance in both specialties. Google Translate and UD Talk exhibited substantially higher similarity (2131/2450, 87%) to each other than to ChatGPT in translated outputs (119/2450, 4.9% to 122/2450, 5%, respectively; *P*<.001).

**Conclusions:**

In Chinese-to-Japanese translations of outpatient medical consultations, ChatGPT demonstrated higher accuracy and user satisfaction than Google Translate and UD Talk. These findings indicate that artificial intelligence–assisted translation may support multilingual clinical communication when used as a complementary aid alongside professional interpreter services.

## Introduction

When health care providers and patients do not share a common language, language barriers arise, hindering effective communication. These communication challenges have major implications for health care costs, quality of care [1], and equity in access to health care services [2]. From ethical and equity-based perspectives, health care professionals are obligated to provide high-quality, human rights–based care to all patients regardless of language differences [3]. Patients with limited proficiency in the dominant language of the health care system often face barriers to accessing care [4] and experience poorer health outcomes than native speakers [5,6].

According to the International Medical Interpreters Association, an interpreter’s primary task is defined as transforming a message from a source language into an equivalent message in a target language such that it elicits the same response in the listener as the original message [7]. Prior studies have demonstrated that the involvement of professional interpreters ensures more favorable patient outcomes than other modes of interpretation [8-10]. Professionally trained on-site interpreters also provide more accurate and reliable verbatim translation than untrained individuals, such as patients’ family members [8]. Qualitative studies involving interpreters, health care professionals, and patients have indicated that interpreters support communication beyond direct translation, such as by facilitating cultural understanding, clarifying medical information, and helping patients navigate the health care process [9,10]. Despite their importance in promoting equitable health care communication, professional interpreters remain underused in routine clinical practice because of structural and logistical barriers such as limited availability, time constraints, and administrative barriers. Patients often experience communication difficulties when medical information is not conveyed in their preferred language, particularly in outpatient settings, where interpreter services are less consistently available [11,12].

Google Translate is a widely used machine translation service and has been frequently examined in academic research on translation accuracy. Since 2016, Google Translate has primarily relied on Google Neural Machine Translation, a deep learning–based neural network that translates complete sentences rather than fragmented text segments, as was characteristic of earlier statistical machine translation methods. Although this change has improved overall translation quality, several studies evaluating Google Translate in medical contexts have reported variable accuracy and potential risks associated with translation errors, raising concerns regarding its suitability for unsupervised clinical use [13-16]. Studies evaluating Google Translate in medical contexts have reported limited accuracy. When counseling information for the 100 most commonly prescribed medications in the United States was translated from English into other languages, accuracy rates were only 54% for Arabic, 77% for Chinese, and 38% for Spanish. Among these errors, 29% were considered clinically significant or potentially life-threatening [17].

UD Talk [18] is a Japanese subtitle application that uses speech recognition technology to convert spoken language into real-time captions, typically within 1 or 2 seconds. The system integrates speech recognition and machine translation functions, and its multilingual translation component is primarily based on Google Translate technology. UD Talk is designed mainly for live interpretation scenarios, whereas Google Translate and ChatGPT are more commonly used for text-based translation tasks.

Advances in large language models (LLMs) have opened new avenues for automated medical translation, particularly in multilingual clinical settings. Artificial intelligence (AI) has become a major focus of research because it simulates human intelligence and cognitive processing to address complex problems [19]. When trained on large and specialized datasets, AI systems can learn linguistic patterns and respond to contextual challenges [20,21]. ChatGPT is an LLM introduced in 2022. Since then, it has been promoted as an advanced language-processing tool. Using extensive datasets, ChatGPT effectively captures linguistic nuance and contextual meaning across multiple languages, predicts word sequences based on context, and generates novel sentences that resemble natural human language. These outputs may be contextually appropriate and extend beyond previously observed text patterns [22].

This study aimed to compare 3 commonly used Chinese-to-Japanese translation tools (ChatGPT, Google Translate, and UD Talk) in real-world medical consultations. Using authentic cardiopulmonary outpatient dialogues, we evaluated the performance of these tools in terms of translation accuracy, error patterns, fluency, contextual appropriateness, user satisfaction, and clinical usability. The analysis further clarified the feasibility and limitations of AI-assisted translation tools for clinical communication and interpreter training.

## Methods

### Study Design

This single-center prospective observational study was conducted in the cardiology and pulmonology outpatient clinics at Fu Jen Catholic University Hospital, Taiwan, between December 2024 and November 2025.

### Ethical Considerations

This study adhered to the principles of good clinical practice and the Declaration of Helsinki. The study protocol was approved by the institutional review board of Fu Jen Catholic University Hospital (approval FJUH113419) and was prospectively registered at ClinicalTrials.gov (identifier: NCT06934031). Written informed consent was obtained from all participants before taking part.

### Study Cohort

Patients were eligible for enrollment if they attended the cardiology or pulmonology outpatient clinics at Fu Jen Catholic University Hospital between December 2024 and November 2025. Patients were excluded if the attending physician determined that they were unable to communicate effectively because of medical or cognitive conditions or if they declined to participate.

### Data Collection and Translation

**Figure 1:**
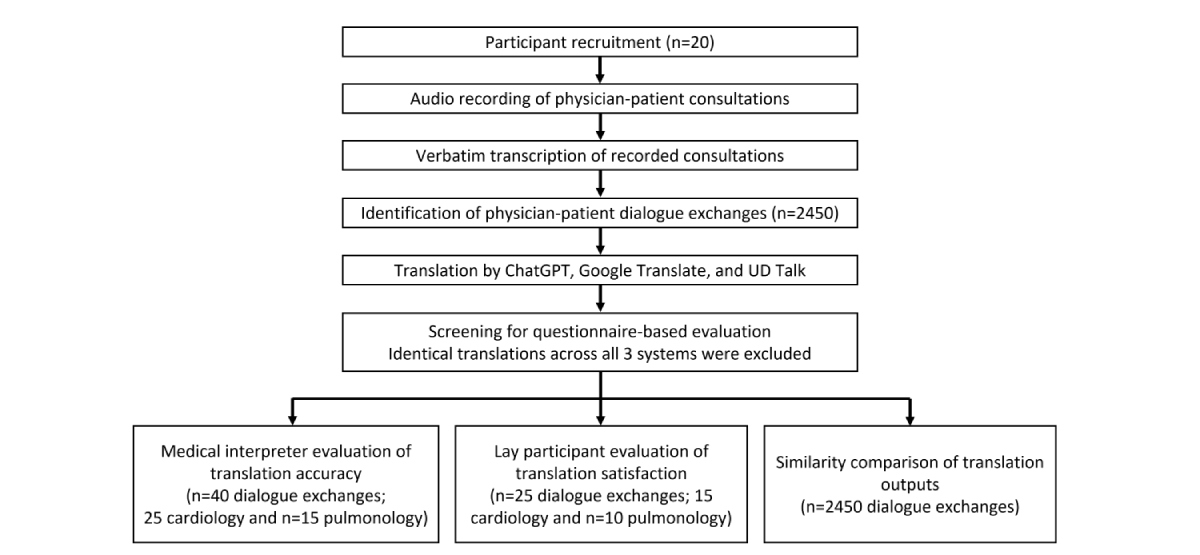
Study workflow.

Audio recordings of physician-patient consultations were collected in the cardiology and pulmonology outpatient clinics. All recorded conversations were transcribed verbatim for analysis. To ensure data confidentiality and protect patient privacy, all personally identifiable information was removed before translation, including names, identification numbers, and any other information that could reveal patient identity (Figure 1).

To preserve real-world clinical communication, transcripts were retained in their original spoken Chinese form without linguistic modification or correction. Transcript volume was quantified using the number of physician-patient dialogue exchanges per outpatient consultation. A dialogue exchange was defined as each individual utterance occurring during the outpatient consultation. Each utterance was separately recorded, transcribed, translated, and labeled according to speaker identity, including physicians, patients, accompanying family members, and clinical staff.

For translation, ChatGPT (GPT-4o; OpenAI) and Google Translate (accessed between April 2025 and September 2025) were used by directly inputting the verbatim transcripts into each system. For ChatGPT translation, standardized prompt instructions were applied before each translation task. The prompts specified speaker identities (D=physician; P=patient; F=family member; S=nurse or other staff member; PF=possible patient or family member), identified the consultation setting as either cardiology or pulmonology outpatient consultation, instructed the model to translate the dialogue into Japanese, and requested translation output only without additional conversational responses or recommendations. Because routine outpatient consultations are inherently conversational and unstructured, no additional linguistic modification, dialogue restructuring, or contextual standardization was applied beyond the original speaker labels to preserve a realistic clinical communication environment as much as possible. In contrast, UD Talk (version 3.19; Shamrock Records; accessed between April 2025 and September 2025) was used in a speech-based manner: a trained research assistant read the original Chinese transcripts aloud, and the spoken input was translated in real time. To reduce variability in speech delivery, all UD Talk inputs were provided by the same research assistant throughout the study.

After translation by all 3 systems, an investigator (CHH) reviewed the outputs and selected dialogue exchanges for questionnaire-based evaluation. Dialogue exchanges with completely identical translated outputs across all 3 systems were excluded to facilitate meaningful comparison across translation systems.

### Evaluation

Translation performance was evaluated from 2 complementary perspectives: assessment of translation accuracy by experts (medical interpreters) and assessment of translation satisfaction by lay participants. A semistructured evaluation format was used, allowing for optional open-ended comments for each item. Each selected dialogue exchange was independently evaluated by 8 medical interpreters and 14 lay participants. Evaluations were conducted using a 6-point Likert scale, with end points ranging from 0 (lowest) to 5 (highest).

For expert evaluation, medical interpreters with formal Chinese-to-Japanese medical interpretation training and at least 2 years of clinical practice experience assessed the accuracy of the translated content. Translation accuracy was defined as the extent to which the translated content conveyed the intended meaning of the original physician-patient communication within the clinical context of the consultation. Interpretation of intended meaning was determined by the research team based on contextual understanding and professional clinical knowledge. Ratings were based on the interpreters’ professional judgment, recognizing that interpreters may prioritize different aspects of translation accuracy, such as semantic fidelity, medical correctness, and clinical appropriateness. Optional qualitative comments could be provided to explain scoring decisions when necessary.

For lay participant evaluation, Japanese individuals residing in Taiwan for at least 2 years rated their overall satisfaction with the translated content. Satisfaction ratings were based on perceived clarity, ease of understanding, and practical usability from the user’s perspective in a clinical communication setting. In addition, both medical interpreters and lay participants completed a separate postevaluation perception survey regarding key components of medical interpretation quality and preferred translation tools.

All evaluation materials were derived from authentic physician-patient consultations and were presented in their original spoken Chinese form. To preserve real-world clinical communication, no linguistic modifications were made to the transcripts before evaluation, retaining incomplete sentences, colloquial expressions, and ambiguous phrasing.

### Statistical Analysis

All statistical analyses were performed using SPSS (version 25; IBM Corp). Descriptive statistics were used to summarize participant characteristics and dialogue exchange volumes. Continuous variables are presented as means with SDs or medians with IQRs, and categorical variables are presented as numbers and percentages.

Translation accuracy and satisfaction scores were measured on ordinal Likert scales; therefore, nonparametric statistical methods were used. Differences in translation accuracy and satisfaction scores among the 3 translation systems were assessed using the Friedman test. Subgroup analysis by outpatient specialty (cardiology vs pulmonology) was conducted using the Wilcoxon rank-sum test. Similarity among the translation outputs of the 3 systems was analyzed at the level of physician-patient dialogue exchanges using the Cochran *Q* test. Interrater reliability for medical interpreter–rated accuracy and lay participant–rated satisfaction was evaluated using the Cronbach α coefficient. A 2-tailed *P* value of less than .05 indicated statistical significance.

## Results

This study included 20 outpatient consultations from 10 cardiology patients and 10 pulmonology patients. The mean number of physician-patient dialogue exchanges per outpatient consultation was 122.5 (SD 41.9). The mean dialogue volume was lower in cardiology consultations than in pulmonology consultations (107.2, SD 37.0 vs 137.8, SD 42.7; Table 1). Eight medical interpreters participated in the expert evaluation. Among them, 2 were native Japanese speakers, 3 had 5 to 10 years of professional experience, 2 had 11 to 20 years of professional experience, and 3 had more than 20 years of professional experience. A total of 14 lay participants were recruited for satisfaction evaluation, all of whom were native Japanese speakers. Most lay participants (13/14, 92.9%) had resided in Taiwan for more than 10 years, including 69.2% (9/13) with 11 to 20 years of residence and 30.8% (4/13) with more than 20 years of residence. Interrater reliability analysis revealed high internal consistency for both medical interpreter and lay participant evaluations. Cronbach α coefficients were 0.942 for medical interpreter–rated translation accuracy and 0.966 for lay participant–rated translation satisfaction, indicating excellent agreement among raters in both groups. Following screening for questionnaire-based evaluation, 40 dialogue exchanges (cardiology: n=25, 62.5%; pulmonology: n=15, 37.5%) were selected for medical interpreter evaluation (Multimedia Appendix 1), and 25 dialogue exchanges (cardiology: n=15, 60%; pulmonology: n=10, 40%) were selected for lay participant evaluation. Dialogue exchanges with completely identical translated outputs across all 3 systems were excluded to facilitate meaningful comparison across translation systems (Multimedia Appendix 2).

Medical interpreter–rated translation accuracy significantly varied across the 3 translation systems (Table 2). Overall, ChatGPT was the most accurate tool, with a median score of 5.0 (IQR 4.0-5.0), whereas Google Translate and UD Talk had substantially lower median accuracy scores (2.0 for both, IQR 1.0-3.0; *P*<.001). This pattern was consistent between the outpatient specialties. ChatGPT maintained median accuracy scores of 5.0 (IQR 4.0-5.0) in both cardiology and pulmonology consultations (*P*<.001 in all cases). Lay participant–rated translation satisfaction also varied significantly among the 3 systems. ChatGPT received the highest satisfaction ratings, with a median score of 5.0 (IQR 4.0-5.0), whereas Google Translate and UD Talk both yielded median satisfaction scores of 2.0 (IQR 1.0-3.0; *P*<.001). Similarly, ChatGPT achieved the highest satisfaction ratings in both cardiology and pulmonology consultations (median 5.0, IQR 4.0-5.0; *P*<.001 in all cases).

Representative qualitative error examples are shown in Table 3. These examples illustrate clinically relevant translation issues observed in Google Translate and UD Talk outputs, including literal translation, incomplete conveyance of clinical meaning, and potentially misleading phrasing. In these cases, ChatGPT provided translations that were more contextually appropriate and clinically coherent.

**Figure 2:**
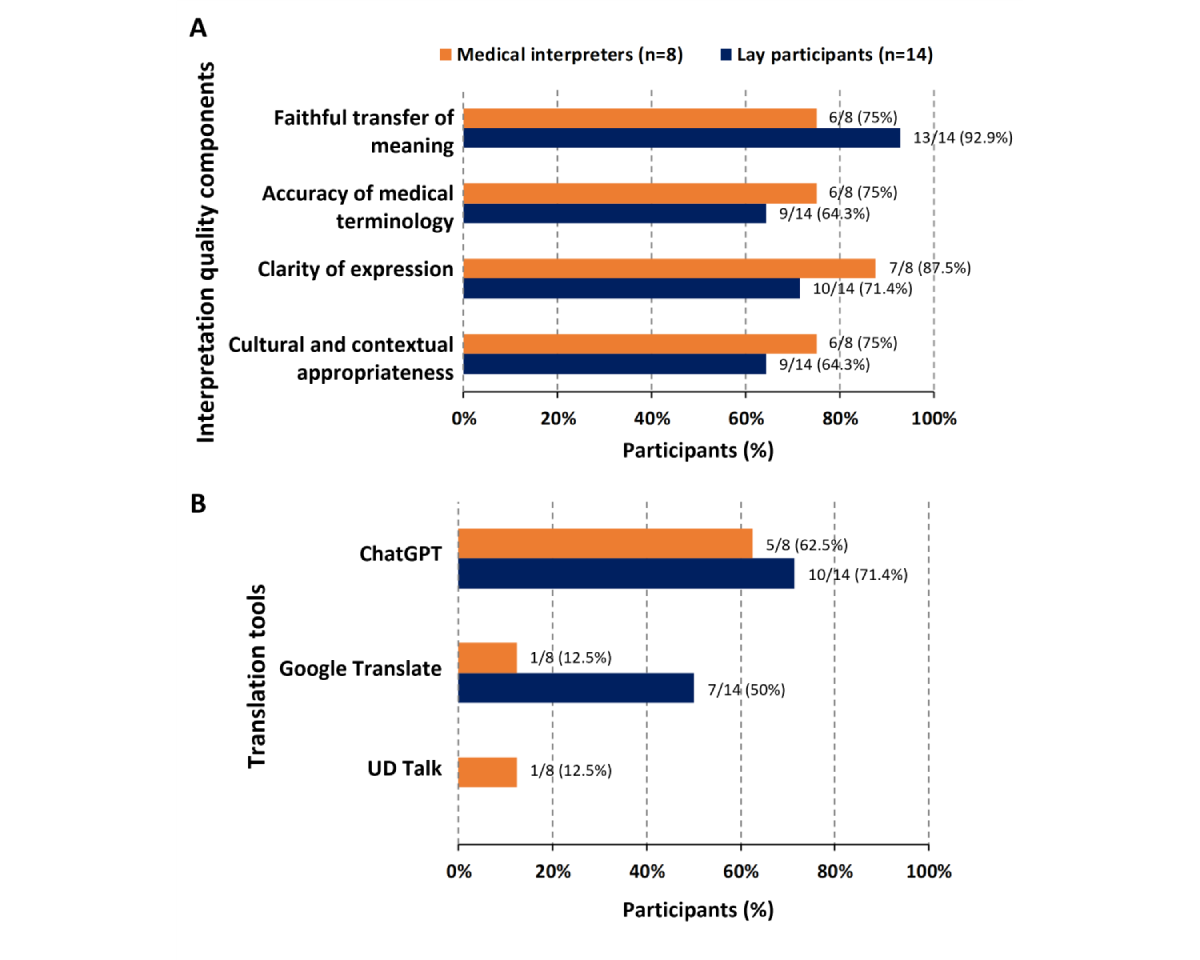
Perceptions of medical interpretation among medical interpreters and lay participants: (A) essential components of medical interpretation accuracy and translation satisfaction as perceived by medical interpreters and lay participants and (B) preferred translation tools of medical interpreters and lay participants.

Perceptions of medical interpretation quality and preferred translation tools were assessed among medical interpreters and lay participants after completion of the evaluation (Figure 2). Clarity of expression was most frequently highlighted as a key component of translation accuracy by medical interpreters (87.5%), followed by faithful transfer of meaning, accuracy of medical terminology, and cultural and contextual appropriateness (each cited by 75% of all interpreters). Faithful transfer of meaning was most frequently highlighted as a key component of translation satisfaction by lay participants (13/14, 92.9%), followed by clarity of expression (10/14, 71.4%). Accuracy of medical terminology and cultural and contextual appropriateness were each identified by 64.3% (9/14) of all lay participants as key components (Figure 2A). Regarding preferred translation tools, ChatGPT was most frequently recommended by both medical interpreters (62.5%) and lay participants (10/14, 71.4%). Google Translate was selected by 50% (7/14) of all lay participants but only 12.5% (1/8) of all medical interpreters, whereas UD Talk was rarely preferred by either group (Figure 2B).

Similarity analysis revealed substantial overlap between Google Translate and UD Talk at the level of physician-patient dialogue exchanges (Table 4). Overall, 87% (2131/2450) of all exchanges produced identical translations between these 2 systems. In contrast, identical outputs with those of ChatGPT were observed in only 4.9% (119/2450) to 5% (122/2450) of all exchanges (*P*<.001). When stratified by outpatient specialty, the similarity between Google Translate and UD Talk remained high but varied by setting. In cardiology consultations, 74.5% (799/1072) of all dialogue exchanges yielded identical translations between Google Translate and UD Talk, but similarity with ChatGPT was low (29/1072, 2.7% and 31/1072, 2.9%, respectively; *P*<.001). In pulmonology consultations, the similarity between Google Translate and UD Talk increased to 96.7% (1332/1378), but similarity with ChatGPT remained low (90/1378, 6.5% and 91/1378, 6.6%, respectively; *P*<.001).

Subgroup analysis revealed higher translation accuracy and satisfaction in pulmonology consultations than in cardiology consultations for both Google Translate and UD Talk (Multimedia Appendix 3). In contrast, ChatGPT had consistently high accuracy and satisfaction in both specialties, with a median score of 5.0 (IQR 4.0-5.0) for both. No significant differences were observed between cardiology and pulmonology consultations in accuracy (*P*=.25) or satisfaction (*P*=.26) for ChatGPT-translated content.

**Table 1:** Characteristics of outpatient consultation recordings, medical interpreters, and lay participants.

Variable					Values
**Dialogue exchanges per outpatient visit, mean (SD)**					
	Overall (n=20 outpatient consultations)			122.5 (41.9)	
	Cardiology outpatient clinic (n=10 outpatient consultations)			107.2 (37.0)	
	Pulmonology outpatient clinic (n=10 outpatient consultations)			137.8 (42.7)	
**Medical interpreters (n=8), n (%)**					
	Native Japanese speakers			2 (25)	
	**Professional experience (y)**				
		5-10	3 (37.5)		
		11-20	2 (25)		
		>20	3 (37.5)		
**Lay participants (n=14), n (%)**					
	Native Japanese speakers			14 (100)	
	**Length of residence in Taiwan (y)**				
		5-10	1 (7.1)		
		11-20	9 (64.3)		
		>20	4 (28.6)		

**Table 2:** Medical interpreter–rated translation accuracy and lay participant–rated translation satisfaction scores for ChatGPT, Google Translate, and UD Talk^a^.

		ChatGPT (0-5), median (IQR)	Google Translate (0-5), median (IQR)	UD Talk (0-5), median (IQR)	*P* value
**Accuracy**					
	Overall (n=320)	5.0 (4.0-5.0)	2.0 (1.0-3.0)	2.0 (1.0-3.0)	<.001^b^
	Cardiology outpatient clinic (n=200)	5.0 (4.0-5.0)	2.0 (0.0-3.0)	2.0 (0.0-3.0)	<.001^b^
	Pulmonology outpatient clinic (n=120)	5.0 (4.0-5.0)	3.0 (1.0-4.0)	2.0 (1.0-4.0)	<.001^b^
**Satisfaction**					
	Overall (n=350)	2.0 (1.0-3.0)	2.0 (1.0-3.0)	5.0 (4.0-5.0)	<.001^b^
	Cardiology outpatient clinic (n=210)	2.0 (1.0-3.0)	2.0 (1.0-3.0)	5.0 (4.0-5.0)	<.001^b^
	Pulmonology outpatient clinic (n=140)	3.0 (1.0-4.0)	3.0 (2.0-4.0)	5.0 (4.0-5.0)	<.001^b^

^a^Eight medical interpreters evaluated 40 dialogue exchanges (cardiology: n=25, 62.5%; pulmonology: n=15, 37.5%) for translation accuracy, and 14 lay participants evaluated 25 dialogue exchanges (cardiology: n=15, 60%; pulmonology: n=10, 40%) for translation satisfaction.

^b^*P*<.001.

**Table 3:** Representative clinically relevant translation errors across the 3 translation systems^a^.

Clinical scenario and source			Dialogue		Expert commentary	
**Cardiology example**						Both Google Translate and UD Talk mistakenly segmented “腎指數” as “kidney+index finger,” producing nonsensical output. ChatGPT correctly interpreted it as “renal function value” (腎機能の数値).
	Original dialogue	Family member: “他的指、腎指數真的是非常不好，真的不能再打針了。”				
	ChatGPT	Family member: “彼の腎機能の数値は本当に悪くて、もう注射は絶対にダメだって言われたんです。”				
	Google Translate	Family member: “彼の指と腎臓の人差し指の状態は本当に悪く、もう注射を受けることができません。”				
	UD Talk	Family member: “彼の指と腎臓の人差し指の状態は本当に悪く、もう注射を受けることは本当に不可能なんです。”				
**Pulmonology example**						Google Translate and UD Talk incorrectly translated the head “heaviness/fogginess” as physical swelling (腫れている). ChatGPT correctly rendered it as “ぼーっとする”—clinically accurate for the patient’s chief concern.
	Original dialogue	Patient: “現在我就擔心這個頭一直脹脹的，不知道什麼⋯⋯”				
	ChatGPT	Patient: “今はこの頭がずっとぼーっとする感じが気になっていて……何が原因なのか分からなくて。”				
	Google Translate	Patient: “頭がずっと腫れているのが心配なんです。どうしてなのかわからないんですけど…”				
	UD Talk	Patient: “頭が腫れている理由が心配です。何が起こっているのか分かりません…”				

^a^Original dialogues were retained verbatim from real outpatient recordings, including disfluencies, repetitions, self-corrections, and incomplete expressions. No linguistic corrections were made.

**Table 4:** Percentage similarity of translation outputs across systems^a^.

	Google Translate vs UD Talk, n (%)	ChatGPT vs UD Talk, n (%)	ChatGPT vs Google Translate, n (%)	*P* value
Overall dialogue exchanges (n=2450)	2131 (87)	122 (5)	119 (4.9)	<.001^b^
Cardiology outpatient clinic dialogue exchanges (n=1072)	799 (74.5)	31 (2.9)	29 (2.7)	<.001^b^
Pulmonology outpatient clinic dialogue exchanges (n=1378)	1332 (96.7)	91 (6.6)	90 (6.5)	<.001^b^

^a^Similarity analyses were conducted at the level of physician-patient dialogue exchanges.

^b^*P*<.001.

## Discussion

### Principal Findings

We analyzed 20 outpatient consultations comprising 2450 physician-patient dialogue exchanges and compared 3 common Chinese-to-Japanese translation tools in terms of their performance in real-world clinical settings. In both cardiology and pulmonology consultations, ChatGPT consistently exhibited higher translation accuracy and user satisfaction than Google Translate and UD Talk, with median scores at the highest level of the rating scale. In contrast, Google Translate and UD Talk had substantially lower scores and exhibited high similarity in translated outputs, indicating shared limitations in handling conversational clinical language. This high similarity may reflect their reliance on conventional machine translation or speech recognition–based processing, which prioritizes literal sentence conversion rather than contextual interpretation.

Our findings suggest that ChatGPT may have potential utility as an AI-assisted translation tool in Chinese-to-Japanese outpatient medical communication. Unlike conventional machine translation systems, which rely primarily on sentence- or phrase-level processing, ChatGPT generates natural and appropriate responses by interpreting broader contextual meaning. This difference in processing may explain the higher translation accuracy and satisfaction observed for ChatGPT, particularly when handling colloquial spoken Chinese and clinically nuanced dialogue. The findings also indicate that ChatGPT may be applicable across different outpatient specialties within the context of this study.

Although subgroup analysis revealed slightly higher translation accuracy and satisfaction scores in pulmonology consultations than in cardiology consultations, ChatGPT maintained high performance in both specialties. Specialty-based differences were noted primarily for Google Translate and UD Talk. This may partly reflect differences in the selected dialogue content. Cardiology exchanges more frequently involved treatment decision-making, procedural planning, medication use, self-paid treatment discussions, cardiac rhythm assessment, diagnostic certificates, and fragmented patient narratives. In contrast, pulmonology exchanges were more often related to symptom confirmation, examination arrangement, medication instructions, and patient education, with more explicit clinical context. These differences may have made pulmonology dialogues easier for conventional translation systems to process. However, ChatGPT exhibited the highest performance in both settings. Therefore, specialty-based differences are unlikely to affect the applicability of ChatGPT in different clinical departments. This consistency indicates that LLM-based translation tools are suitable for use in diverse outpatient specialties without substantial concern for specialty-specific performance limitations.

### Comparison With Previous Literature and Clinical Implications

Despite the promising results, AI-based translation tools should not be regarded as replacements for professional medical interpreters. Prior research has demonstrated that trained interpreters contribute not only to the accuracy of verbal communication but also to the mediation of cultural differences, clarification of medical information, and facilitation of patient engagement [8,11,23-26]. Rao et al [27] reported that, although ChatGPT outperformed Google Translate in Spanish translation, it demonstrated inferior performance in Vietnamese and produced low-quality translations in Russian. In certain languages, both ChatGPT and Google Translate had unacceptably high error rates, indicating that AI-generated translations can be unreliable when used without human oversight. Although AI-based translation tools may perform well in select languages, their broader clinical use requires careful validation and continued human supervision to ensure patient safety and accurate communication. Kong et al [28] evaluated machine translation for patient-specific clinical instructions and reported generally high degrees of sentence-level accuracy but persistent risks of clinically meaningful errors and potential harm, particularly when translations were used without professional oversight or in more complex medical cases.

These findings should be interpreted in light of differences in study design, translation direction, and communication context. Unlike prior studies that often evaluated written medical instructions translated from English into other languages, this study focused on spoken Chinese outpatient dialogue translated into Japanese. Because real-world consultations include incomplete sentences, colloquial expressions, and implicit contextual meaning, translation performance may differ according to both language pair and communication format. This also supports the continued need for professional medical interpreters, who can integrate linguistic accuracy, clinical judgment, cultural mediation, and real-time clarification in complex clinical encounters.

AI-based translation systems may function as supplementary communication tools in settings where professional interpreter services are limited or unavailable, particularly in time-sensitive outpatient care. Future research should investigate how these technologies can be integrated into clinical workflows, interpreter training programs, and patient education to support multilingual communication while maintaining patient safety and quality of care. In practice, such tools may be particularly useful for brief outpatient encounters, preliminary triage communication, and patient education when professional interpreters are not immediately available.

Provision of professional interpreter services is recognized as a global strategy for reducing language barriers in health care. In countries such as Australia, Canada, the United States, and the United Kingdom, language service policies, standards, and guidelines have been established to promote or mandate the use of professional interpreter services in clinical care [29]. Despite these efforts, evidence indicates that the use of professional interpreter services remains suboptimal, whereas reliance on ad hoc interpreters, such as family members, friends, and other untrained individuals, is common [30,31]. In some clinical settings, bilingual health care staff assume the role of interpreters despite lacking formal training. This practice may compromise the quality of interpretation and increase the risk of adverse clinical outcomes [8,32,33]. Therefore, access to professional interpreter services must be enhanced to strengthen communication between health care providers and patients with limited proficiency in the dominant language. A systematic review of 37 studies indicated that reducing language barriers is a key strategy for improving patient safety and minimizing adverse events in hospital care. The review further reported that the use of professional interpreter services substantially improves communication between health care providers and patients and enhances the quality of care for linguistically diverse populations [29]. A systematic review of 29 studies compared 5 interpretation-related situations: involvement of professional interpreters, involvement of ad hoc interpreters, involvement of relational interpreters, involvement of untrained interpreters, and absence of interpreters. The results revealed that the involvement of face-to-face professional interpreters was associated with the highest patient satisfaction and the most effective communication. Moreover, any form of interpretation was determined to be superior to no interpreter use, and relational interpreters represented useful communication resources in certain private clinical settings [26].

### Qualitative Insights From Medical Interpreters

In addition to the quantitative findings, qualitative feedback from medical interpreters provided insights into the strengths and limitations of the translation tools. Interpreters emphasized that accurate transfer of meaning and correct use of medical terminology are essential for clinical translation. Although some lay participants considered minor grammatical imperfections acceptable when the overall meaning remained understandable, interpreters exhibited low tolerance for semantic distortion or incorrect terminology because such errors could lead to clinical misunderstanding. ChatGPT was perceived to handle contextual information and incomplete or colloquial spoken language more effectively than the other systems. Interpreters noted that its translations often clarified implicit meaning and restructured fragmented sentences, which in some cases resembled the output produced by human interpreters. In contrast, Google Translate and UD Talk were frequently described as relying on more literal translation patterns, which resulted in awkward phrasing or incomplete conveyance of clinical meaning.

Interpreters indicated that, although AI-based translation tools performed favorably, they should be used with caution and primarily to support communication rather than to replace professional interpreters, particularly in high-risk clinical situations. Interpreters also noted that the use of AI translation in health care raises substantial legal, ethical, and policy challenges, such as concerns related to data privacy, informed consent, accountability for translation errors, and potential bias in multilingual communication [34]. Therefore, a collaborative human-AI approach was recommended to ensure accuracy, clarity, and patient safety in multilingual clinical communication while addressing these regulatory and ethical concerns.

### Limitations

This study has several limitations. First, the analysis exclusively focused on Chinese-to-Japanese translation; thus, the findings may not be generalizable to other language pairs with different linguistic structures and resource availability levels. Second, although the dataset included 20 outpatient consultations and 2450 dialogue exchanges, the sample size and text volume may still be insufficient to capture the full diversity of real-world clinical communication. Third, consultations in each specialty were conducted by a single physician. Thus, variation in communication styles across physicians within the same specialty was not examined. Differences in physicians’ speech patterns, use of medical terminology, sentence structure, and degree of colloquial expression may influence AI translation performance; thus, potential physician-specific effects cannot be excluded. Finally, translation performance was evaluated using GPT-4o, and the findings may not be generalizable to newer versions of ChatGPT or other LLMs. In the future, studies including multiple language pairs, larger datasets, diverse clinical settings, multiple physicians within each specialty, and updated AI models should be conducted to validate and extend our findings.

### Conclusions

This study demonstrated that ChatGPT achieved higher translation accuracy and user satisfaction than Google Translate and UD Talk in Chinese-to-Japanese translations of real-world outpatient consultations. However, AI-based translation should be implemented as a complementary communication tool rather than a replacement for professional medical interpreters. Careful integration into clinical workflows and continued human oversight remain essential to ensure patient safety, communication accuracy, and equitable multilingual health care delivery.

## Appendix

Multimedia Appendix 1 Instrument for evaluating the medical interpreter–rated accuracy of the translations.

Multimedia Appendix 2 Instrument for evaluating the lay participant–rated satisfaction with the translations.

Multimedia Appendix 3 Subgroup analyses of translation accuracy and satisfaction across translation systems by outpatient specialty.

## Abbreviations

AI: artificial intelligence
LLM: large language model

## Data Availability

The data supporting the findings of this study are included in the supplementary materials.
