# Supplementary material for: Comparative Evaluation of ChatGPT, Google Translate, and UD Talk for Chinese-to-Japanese Translation in Cardiology and Pulmonology Outpatient Consultations: Prospective Observational Study
Source: J Med Internet Res. 2026 Jun 18;28:e93082. doi: 10.2196/93082 (PMC13329329; doi:10.2196/93082)
Supplement: Multimedia Appendix 1 [file jmir_v28i1e93082_app1.docx]

**Multimedia Appendix 1.** Instrument for evaluating the medical interpreter–rated accuracy of the translations.

| **No.** | **Content** | **Rating** | | | | | |
| --- | --- | --- | --- | --- | --- | --- | --- |
|  |  | **0** | **1** | **2** | **3** | **4** | **5** |
| **1** | **D：嘿。哈囉，最近還好嗎？你那個藥吃的怎麼樣？人有沒有不舒服？** |  |  |  |  |  |  |
| 1A | D：やあ。こんにちは。最近調子はどうですか？薬はどうですか？体調が悪い人はいませんか？ |  |  |  |  |  |  |
| 1B | D：やあ、こんにちは。調子はどう？薬は大丈夫？体調は悪い？ |  |  |  |  |  |  |
| 1C | D：やあ、こんにちは。最近はどうですか？あのお薬はちゃんと飲めていますか？体調に変わりはありませんか？ |  |  |  |  |  |  |
| **2** | **P：他說是發作再吃** |  |  |  |  |  |  |
| 2A | P：襲われた時に食べたと言ってました。 |  |  |  |  |  |  |
| 2B | P：攻撃が起こったらそれを取ると言っていました。 |  |  |  |  |  |  |
| 2C | P：発作が起きた時に飲むようにと言われました。 |  |  |  |  |  |  |
| **3** | **F：他的指、腎指數真的是非常不好，真的不能再打針了。** |  |  |  |  |  |  |
| 3A | F：彼の指と腎臓の人差し指の状態は本当に悪く、もう注射を受けることができません。 |  |  |  |  |  |  |
| 3B | F：彼の指と腎臓の人差し指の状態は本当に悪く、もう注射を受けることは本当に不可能なんです |  |  |  |  |  |  |
| 3C | F：彼の腎機能の数値は本当に悪くて、もう注射は絶対にダメだって言われたんです。 |  |  |  |  |  |  |
| **4** | **P：現在我就擔心這個頭一直脹脹的，不知道什麼⋯⋯** |  |  |  |  |  |  |
| 4A | P：頭がずっと腫れているのが心配なんです。どうしてなのかわからないんですけど… |  |  |  |  |  |  |
| 4B | P：頭が腫れている理由が心配です。何が起こっているのか分かりません… |  |  |  |  |  |  |
| 4C | P：今はこの頭がずっとぼーっとする感じが気になっていて……何が原因なのか分からなくて。 |  |  |  |  |  |  |
| **5** | **D：你就跟他說，說醫生說沒開這種的。我可以開一般的診斷書啊，你什麼病，於什麼時候來心臟科門診看診，這個可以。齁，啊但是我、我沒有辦法開說「你適合上班」，這、這我沒辦法開。這樣子。我，我可以開說你適、「宜休養一個月，不要從事太激烈的工作或活動」。你要這樣開嗎？**  **（情境說明：病人請醫生開他可以照常上班的診斷書，以利向公司交待。）** |  |  |  |  |  |  |
| 5A | D：医師はこれを処方していないと言ったと伝えてください。一般的な診断、どんな病気なのか、そしていつ心臓内科の診察を受けるべきかを処方することはできます。大丈夫です。ああ、でも「仕事に行っても大丈夫です」とは処方できません。こういう処方はできません。「1ヶ月間休養し、あまり激しい仕事や活動は控えてください」と処方することはできます。これを処方しますか？ |  |  |  |  |  |  |
| 5B | D：医師から処方箋を出していないと言われたと伝えてください。大まかな診断名、どんな病気なのか、そしていつ心臓科の診察を受けるべきかは書きます。大丈夫です。ああ、でも「仕事に行っても大丈夫です」なんて書けません。こんな風に。「1ヶ月間休養し、あまり激しい仕事や活動控えてください」なんてことは言えます。これ、書きますか？ |  |  |  |  |  |  |
| 5C | D：会社には「医者がそういう証明書は出していない」と伝えてください。私は普通の診断書なら書けますよ。たとえば「○○という病気で、いつ心臓内科を受診した」とか、そういう内容なら大丈夫です。でも「あなたは仕事に適している」とか「勤務可能」といった内容は、私の立場では出せません。私が書けるのは「1ヶ月間の休養が望ましく、激しい仕事や活動は控えるべき」といった内容です。それで診断書を出す形でもよければ、そうしますが、どうしますか？ |  |  |  |  |  |  |
| **6** | **D：（應聲）妳有沒有回過門診？去看過？** |  |  |  |  |  |  |
| 6A | D：(答える) クリニックに戻ったことはありますか？行ったことはありますか？ |  |  |  |  |  |  |
| 6B | D：(応答) クリニックに戻ったことはありますか? クリニックに行ったことはありますか? |  |  |  |  |  |  |
| 6C | D：（うなずきながら）外来には戻って診てもらったことがありますか通院されましたか？ |  |  |  |  |  |  |
| **7** | **D：（應聲）妳有沒有回過門診？去看過？ P：我，有啊。我就一直在看哪。**  **（請評比底線部份的譯文）** |  |  |  |  |  |  |
| 7A | P：はい、私もずっと見ていました。 |  |  |  |  |  |  |
| 7B | P：ええ、私も。ただ見ていたんです。 |  |  |  |  |  |  |
| 7C | P：はい、あります。ずっと通院していますよ。 |  |  |  |  |  |  |
| **8** | **D：那妳有做過心臟，他有給妳做過心臟超音波嗎？**  **（情境說明：醫生詢問病人在其它診所時，有沒有做過心臟超音波檢查）** |  |  |  |  |  |  |
| 8A | D：心臓手術を受けたことはありますか？心臓の超音波検査は受けましたか？ |  |  |  |  |  |  |
| 8B | D：心臓スキャンを受けたことはありますか？超音波検査も受けましたか？ |  |  |  |  |  |  |
| 8C | D：それで、心臓のエコー検査（心臓超音波）は、受けたことがありますか？先生がしてくれましたか？ |  |  |  |  |  |  |
| **9** | **D：沒有，現在健保⋯⋯其實換醫生齁，還是建議自己要帶資料過來。**  **P：嗯。**  **D：因為那樣子才不會搞錯。健保資料很陽春，常常又當機連不上去。**  **（請評比底線部份的譯文）** |  |  |  |  |  |  |
| **9A** | **D：そうすれば間違いがなくなるからです。健康保険の情報は非常に基本的なもので、よくクラッシュして接続できないんです。** |  |  |  |  |  |  |
| **9B** | **D：そうすれば間違いが起こらないからです。健康保険の情報は非常に基本的なもので、頻繁にクラッシュしたり接続できなかったりします。** |  |  |  |  |  |  |
| **9C** | **D：そうすれば間違いも防げますからね。健康保険のデータはけっこう簡易的で、よく接続できなくなったりもしますし。** |  |  |  |  |  |  |
| **10** | **D：（應聲）對對。喔⋯⋯他什麼時候要回甲醫師那邊？喔！他三個月餒，太久了。**  **P & F：誒對。剛剛才⋯⋯**  **D：剛剛才？啊甲醫師有說要追蹤嗎？**  **（請評比底線部份的譯文）** |  |  |  |  |  |  |
| 10A | D：今ですか？A医師は経過観察が必要だと言いましたか？ |  |  |  |  |  |  |
| 10B | D：今？A医師は経過観察したいと言っていましたか？ |  |  |  |  |  |  |
| 10C | D：さっきちょうど？ああ、甲先生はフォローアップ（経過観察）するように言ってましたか？ |  |  |  |  |  |  |
| **11** | **D：那沒問題。那既然你描述的已經很清楚了齁，那，我就幫你排幾個比較重要的檢查。一個叫24小時心電圖，那個抓心率不整。喔它掛在你身上一整天，當天不能洗澡隔天拆掉。那心臟超音波幫你同一個時間一起拍，好不好？啊你什麼時候比較方便？ P：呃，那我、我很好奇，我其實現在是空腹超過8小時，我可以今天。**  **（請評比底線部份的譯文）** |  |  |  |  |  |  |
| 11A | P：えっと、ちょっと気になるんですが、実は8時間以上断食しているんです。 |  |  |  |  |  |  |
| 11B | P：えっと、それでは、私は、とても興味があるのですが、実はもう 8 時間以上断食をしているので、今日はそれができるんです。 |  |  |  |  |  |  |
| 11C | P：ええと、今ちょうど8時間以上何も食べていなくて、空腹なんですけど……今日でも検査は可能ですか？ |  |  |  |  |  |  |
| **12** | **P：胃、胃在晚一點的時候，還是我喝茶喝咖啡的原因？**  **D：你說會緊緊的嗎？**  **（請評比底線部份的譯文）** |  |  |  |  |  |  |
| 12A | P：胃、一日の終わりに胃が痛くなる、それともお茶やコーヒーを飲むからでしょうか? |  |  |  |  |  |  |
| 12B | P：それは胃のせいでしょうか、それとも夜遅くに飲むお茶やコーヒーのせいでしょうか? |  |  |  |  |  |  |
| 12C | P：胃が…遅い時間になるとちょっと…。それとも、お茶とかコーヒーを飲むせいですか？ |  |  |  |  |  |  |
| **13** | **D：聽一下齁。【聽診】OK，心臟聽起來很好。 P：好。就是⋯⋯，反正昨天因為突然出現差不多160下，就是100⋯⋯，然後安靜的時候120，我想説跟平常不一樣**  **（請評比底線部份的譯文）** |  |  |  |  |  |  |
| 13A | P：はい。つまり、昨日は突然現れたので160回くらい、つまり100回くらい…、そして静かになった時に120回くらいでした。いつもと違っていたと思います。 |  |  |  |  |  |  |
| 13B | P：わかりました。ただ…、とにかく昨日は突然現れたので160回くらい、つまり100回…、そして静かだったのに120回くらいだったので、いつもと違うなと思います。 |  |  |  |  |  |  |
| 13C | P：はい。えっと……とにかく昨日は突然心拍数が160くらいまで上がって、安静にしているときでも120くらいあって、いつもと違うなと思って。 |  |  |  |  |  |  |
| **14** | **D：好了。我跟你說，我這一次齁，也不要開太久啦，因為你胃還在不舒服，而且有一些檢查也還沒做完整。我們一個月回來好不好？***  **（情境說明：醫師開藥）** |  |  |  |  |  |  |
| 14A | D：分かりました。実は、お腹がまだ痛くて、検査もまだ終わっていないので、今回はあまり長く開けたままにしないようにしています。1ヶ月後にまた来ていただけますか？ |  |  |  |  |  |  |
| 14B | D：分かりました。実は、今回はあまり長く空けておくつもりはありません。お腹がまだ痛くて、検査もまだ終わっていないので。1ヶ月後にまた来ていただけますか |  |  |  |  |  |  |
| 14C | D：じゃあ今回は、あまり間隔を空けずにしておきましょう。胃の調子もまだ良くないし、いくつかの検査もまだ終わってないですからね。1ヶ月後にもう一度来てもらうということで、どうですか？ |  |  |  |  |  |  |
| **15** | **D：齁。好。那就這樣子囉。齁，那我們就先檢查完整，啊你也測一下血壓。下次把藥物帶過來，那我們確定一下，看⋯到時候的計劃怎麼定，這樣好不好？** |  |  |  |  |  |  |
| 15A | D：ああ。分かりました。それで終わりです。あ、まずは精密検査をしましょう。血圧も測れますよ。次回薬を持ってきてください。それで確認して…どう計画を立てるか…それでいいですか？ |  |  |  |  |  |  |
| 15B | D：ああ。分かりました。それで終わりです。あ、まずは精密検査をしましょう。血圧も測れますよ。次回薬を持ってきてください。それで確認して、どうするか決めましょう。よろしいですか？ |  |  |  |  |  |  |
| 15C | D：うん、じゃあ今日はこれで終わりにしましょう。まずはしっかり検査をして、あなたも血圧を測ってください。次回来るときにはお薬も持ってきてくださいね。それで、今後の治療方針を一緒に確認しましょう。いいですか？ |  |  |  |  |  |  |
| **16** | **D：胃藥**  **P：應該可以不開了。**  **D：可以不用了啦齁。好**  **（請評比底線部份的譯文）** |  |  |  |  |  |  |
| 16A | D：大丈夫ですよ。 |  |  |  |  |  |  |
| 16B | D：大丈夫だよ。OK。 |  |  |  |  |  |  |
| 16C | D：もう必要ないですね。はい。 |  |  |  |  |  |  |
| **17** | **P：啊我來做檢查回去跟我兒子討論喔，「啊媽媽齁，要跟甲醫師說，又要用自費的，**  **P：是貴一點，那我可以撐好幾次**  **P：我不要一直進醫院，我不要，**  **P：我會、我會怕啦。」**  **(情境說明：病人說明跟兒子討論時，有說打算向醫師要求裝自費的心臟支架）** |  |  |  |  |  |  |
| 17A | P：検診に行って、息子と相談します。「お母さん、A先生に伝えなきゃいけないし、費用も自分で払わなきゃいけないんだ」  P：少し高いですが、何回か使えます。  P：いつも病院に行きたくないんです。行きたくないんです。  P：怖いですね。 |  |  |  |  |  |  |
| 17B | P：検査に行って、息子と相談します。「あら、お母さん、A先生に伝えて、自分でお金を払わないといけないの」  P：少し高いですが、何回か使えます。  P：いつも病院に通いたくないんです、そんなことはしたくないんです。  P：そうでしょうね、怖いですから。」 |  |  |  |  |  |  |
| 17C | P：それで検査を受けてから息子と相談したんです。「お母さんね、また甲先生に自費のステントをお願いしたいって言ってるんだけど…」って。  P：ちょっと高いけど、それで何回も持つならいいかなって思って。  P：もう何度も入院したくないんです。いやなんです。  P：怖いんです、本当に。 |  |  |  |  |  |  |
| **18** | **D：我們大概就是一兩年齁，就讓你再穩定一兩年。那個跑步心電圖跟心臟超音波都要再追蹤。你跑步...有辦法跑步啦齁？** |  |  |  |  |  |  |
| 18A | D：おそらく1、2年待って、その後さらに1、2年かけて状態を安定させます。心電図と心臓超音波検査を再度記録します。...逃げる方法はあるんですか？ |  |  |  |  |  |  |
| 18B | D：停滞期間はおそらく1、2年程度でしょうから、あと1、2年は安定期に入るまで待ちましょう。ランニング心電図と心臓超音波検査を再度記録します。走ってください。...逃げる方法はあるんですか？ |  |  |  |  |  |  |
| 18C | D：だいたい1〜2年ごとに、安定していれば心電図（運動負荷付き）と心臓エコーの両方を再検査します。運動は…...走ったりするのは大丈夫そうですか？ |  |  |  |  |  |  |
| **19** | **P：因為昨天⋯下午嘔吐， P：然後吐完以後就、就⋯⋯手跟頭還有點麻。 D：（應聲） P：然後越來越，變成說心臟也、有麻，然後會一直縮、縮緊的感覺。**  **（請評比底線部份的譯文）** |  |  |  |  |  |  |
| 19A | P：昨日の午後に吐いてしまったから。  P：吐いた後も、手と頭が少し痺れていました。  D：(応答)  P：その後、症状はどんどんひどくなり、心臓が麻痺したように感じ、縮み、締め付けられるような感じになりました。 |  |  |  |  |  |  |
| 19B | P：昨日の午後に吐いてしまったから。  P：吐いた後も、手と頭が少し痺れていました。  D：(応答)  P：それから症状はどんどんひどくなり、心臓が麻痺したように感じ始め、縮み、締め付けられるような感じが続きました。 |  |  |  |  |  |  |
| 19C | P：昨日の午後、嘔吐したので…。  P：吐いた後に、手と頭がちょっとしびれる感じがありました。  D：（はいはい）  P：それでだんだん、心臓にもしびれるような感じがして、ずっと締めつけられるような感覚になってきました。 |  |  |  |  |  |  |
| **20** | **D：妳那樣發作過幾次？ P：很多次。 D：很多次喔。 P：只是最⋯⋯，這是比較最近的，的這兩次的，就是3月16**  **（請評比底線部份的譯文）** |  |  |  |  |  |  |
| 20A | P：ただ、一番最近の…これが一番最近の 2 つのもので、3 月 16 日のものでした。 |  |  |  |  |  |  |
| 20B | P：でも、一番最近の 2 回は 3 月 16 日でした。 |  |  |  |  |  |  |
| 20C | P：ただ、最近のは特にこの2回で……3月16日です。 |  |  |  |  |  |  |
| **21** | **D：哦哦。現在比較沒有咳？**  **P：現在⋯⋯咳嗽，沒有。**  **（請評比底線部份的譯文）** |  |  |  |  |  |  |
| 21A | D：ああ、咳は治まりましたか？ |  |  |  |  |  |  |
| 21B | D：あらまあ。咳は治まりましたか？ |  |  |  |  |  |  |
| 21C | D：ああ、今はあまり咳は出ていませんか？ |  |  |  |  |  |  |
| **22** | **D：因為有一些，誒⋯⋯有一些藥我們還是要等確切檢查結果出來了，再開比較好。** |  |  |  |  |  |  |
| 22A | D：ええと、処方する前に正確な試験結果が出るまで待たなければならない薬がいくつかあるからです。 |  |  |  |  |  |  |
| 22B | D：ええと、いくつかの薬は、処方する前に正確な検査結果が出るまで待たなければなりません。 |  |  |  |  |  |  |
| 22C | D：というのも、いくつかのお薬については、やはり検査結果がはっきり出てから処方した方がいいので。 |  |  |  |  |  |  |
| **23** | **D：這樣好了啦，我開一個緊急的吸劑給你。緊急的就是說如果你真的比較喘的時候可以吸。藍色的，你有沒有拿過？** |  |  |  |  |  |  |
| 23A | D：わかりました。緊急用吸入器を処方します。緊急用というのは、本当に息切れがひどい時に使うものです。青い色の吸入器ですね。使ったことはありますか？ |  |  |  |  |  |  |
| 23B | D：わかりました。緊急用の吸入器を処方します。息切れがひどい時に使うものです。青い色のものです。使ったことはありますか？ |  |  |  |  |  |  |
| 23C | D：ではこうしましょう。緊急用の吸入薬をひとつ出しておきますね。本当に息苦しくなったときに使えるものです。青い吸入薬なんですが、使ったことありますか？ |  |  |  |  |  |  |
| **24** | **D：（安排檢查）看看妳有沒有氣管過敏啊等等，**  **P：嗯。**  **D：好不好？齁。那妳現在就要吃藥嗎？還是等我們檢查出來了，確定什麼問題再來吃藥？**  **（請評比底線部份的譯文）** |  |  |  |  |  |  |
| 24A | D：わかりましたか？痛いですね。今すぐ薬を飲まないといけないですか？それとも、症状が確認できるまで待った方がいいですか？ |  |  |  |  |  |  |
| 24B | D：わかりましたか？痛いですね。今すぐ薬を飲まないといけないですか？それとも、検査して何が問題なのかわかるまで待った方がいいですか？ |  |  |  |  |  |  |
| 24C | D：いいですか？じゃあ今すぐお薬を飲みたいですか？それとも検査して原因がはっきりしてから薬を使いたいですか？ |  |  |  |  |  |  |
| **25** | **P：那檢查是現在檢查還是？** |  |  |  |  |  |  |
| 25A | P：今検査を受けたほうがいいですか? |  |  |  |  |  |  |
| 25B | P：今チェックを受け取ってもいいですか? |  |  |  |  |  |  |
| 25C | P：検査は今すぐ行うんですか？それとも……？ |  |  |  |  |  |  |
| **26** | **D：那妳是做X光還是低輻射電腦斷層？**  **P：低輻射。**  **D：做低輻射啦。**  **P：對。**  **D：我幫妳看一下。**  **P：然後我在兩年前有在臺安做。但，臺安那時候是說⋯⋯。在臺安做⋯⋯**  **（請評比底線部份的譯文）** |  |  |  |  |  |  |
| 26A | P：それで2年前に泰安でやったんですよ。でもその時、泰安は…って言ったんですよ。 |  |  |  |  |  |  |
| 26B | P：それから2年前に泰安でやりました。でもその時、泰安は…泰安で働いてるって…って言ってたんです。 |  |  |  |  |  |  |
| 26C | P：それから、2年前にタイアン病院で受けたことがあります。でも、そのときタイアンでは…タイアンで検査を受けたときは… |  |  |  |  |  |  |
| **27** | **F：喔～。沒有，因為那邊寫，你寫6小⋯⋯，那藥袋的明細是寫，6小時吃一次是2顆。** |  |  |  |  |  |  |
| 27A | F：ああ。いや、あそこに書いてあるから、6時間って書いてあるよね…薬袋には6時間ごとに2錠ずつ服用って書いてある。 |  |  |  |  |  |  |
| 27B | F：ああ。いや、あそこに書いてあるから、6時間って書いてあるじゃないですか。薬袋には6時間ごとに2錠ずつ服用するように書いてあるんですよ。 |  |  |  |  |  |  |
| 27C | F：ああ〜。でも、そっちに書いてあるのは、6時間ごとに2錠って書いてあるんですよ |  |  |  |  |  |  |
| **28** | **D：然後我們三個月拿一次藥，妳還是要吸藥啦，** |  |  |  |  |  |  |
| 28A | D：それから、3か月に1回薬を服用することになりますが、その薬は引き続き服用しなければなりません。 |  |  |  |  |  |  |
| 28B | D：それから、3か月に1回薬を服用することになりますが、引き続き薬を服用しなければなりません。 |  |  |  |  |  |  |
| 28C | D：それから、お薬は3ヶ月ごとにもらって、吸入はちゃんと続けてくださいね。 |  |  |  |  |  |  |
| **29** | **D：都沒有齁？那妳最近有感冒嗎？**  **P：誒⋯⋯沒有餒。**  **D：都沒有？**  **（請評比底線部份的譯文）** |  |  |  |  |  |  |
| 29A | D：どれもないんですか？ |  |  |  |  |  |  |
| 29B | D：無しですか？ |  |  |  |  |  |  |
| 29C | D：全くなかったんですね？ |  |  |  |  |  |  |
| **30** | **D：嗯～。妳這個禮拜五有時間來做檢查嗎？還是下禮拜？** |  |  |  |  |  |  |
| 30A | D：うーん。今週の金曜日か来週の健康診断に来られる時間はありますか？ |  |  |  |  |  |  |
| 30B | D：うーん。今週の金曜日、それとも来週の検診は受けられますか？ |  |  |  |  |  |  |
| 30C | D：うーん…。今週の金曜日に検査に来る時間はありますか？それとも来週のほうがいいですか？ |  |  |  |  |  |  |
| **31** | **P：嘿對！有吃藥喔！我又跳到140幾，胸口很痛。然後我又吞了那個心律的藥，就比較好了，因為我已經坐在車上了，我沒轍了。坐在遊覽車上，然後我就吞了有，有慢慢的好一點。** |  |  |  |  |  |  |
| 31A | P：はい！薬を飲みました！心拍数が140まで上がって胸が痛くなりました。それで心拍数を下げる薬を飲みました。もう車に乗っていて他に選択肢がなかったので、その方が効きました。ツアーバスに乗っている時に薬を飲んだら、徐々に良くなっていきました。 |  |  |  |  |  |  |
| 31B | P：はい！薬を飲みました！心拍数が140まで上がって胸が痛くなりました。それで心拍数を下げる薬を飲みました。もう車に乗っていて他に選択肢がなかったので、その方が効きました。ツアーバスに乗っている時に薬を飲んだら、徐々に良くなっていきました。 |  |  |  |  |  |  |
| 31C | P：そうそう！薬はちゃんと飲みましたよ！でも心拍が140台まで上がって、胸がすごく痛くなって……。それでまた不整脈の薬を飲んだら、少し落ち着いてきました。もうバスに乗っている最中だったので、どうしようもなかったんです。観光バスの中で薬を飲んで、少しずつ良くなっていきました。 |  |  |  |  |  |  |
| **32** | **D：沒關係，再做一次。我們看看做兩次能不能抓到那種比較不好的心律不整。因為妳這個心律不整，不是、不是那種⋯⋯單純多一條電線的。這種可能要燒⋯⋯，要看。大概一半的機會是多一條電線的，另外一半的機會是心臟狀況不好，它亂跳一些比較有致命性的心律。總之先檢查，好不好？** |  |  |  |  |  |  |
| 32A | D：大丈夫です。もう一度やりましょう。二度やって、もっとひどい不整脈を見つけられるかどうか見てみましょう。あなたの不整脈は…ただ余分なワイヤーが入っているだけの不整脈ではありません。この不整脈は火傷する恐れがあるので…診なければなりません。余分なワイヤーが入っている可能性は約50%で、残りの50%は心臓の状態が悪く、より危険なリズムで鼓動している可能性です。とにかく、まずは確認しましょう、いいですか？ |  |  |  |  |  |  |
| 32B | D：大丈夫です。もう一度やりましょう。二度やって、もっとひどい不整脈を見つけられるかどうか見てみましょう。あなたの不整脈は…ただ余分なワイヤーが入っているだけの不整脈ではありません。この不整脈は火傷する恐れがあるので…診なければなりません。余分なワイヤーが入っている可能性は約50%で、残りの50%は心臓の状態が悪く、より危険なリズムで鼓動している可能性です。とにかく、まずは確認しましょう、いいですか？ |  |  |  |  |  |  |
| 32C | D：大丈夫です、もう一度やってみましょう。今回もう一度やることで、その“あまりよくないタイプ”の不整脈を捉えられるかもしれません。あなたの不整脈は、単に電気の経路が1本多いというだけの単純なタイプではないように思います。こういったケースでは、場合によってはアブレーション（焼灼）が必要になるかもしれませんが、まずは詳しく調べないといけません。だいたい半分のケースでは余分な電気の経路が原因ですが、もう半分のケースでは心臓そのものの状態が悪くて、危険な不整脈を起こしている可能性もあります。とにかく、まずは検査をしてはっきりさせましょう。いいですか？ |  |  |  |  |  |  |
| **33** | **D：好我跟妳說齁，我先幫妳排檢查。排兩個檢查，一個叫做24小時心電圖，一個叫心臟超音波。妳等一下兩張單子都要給他們。那當然如果他機器要今天早上一定要裝，妳⋯⋯就去吃個飯，下午過來做心臟超音波，裝著我也可以做，齁，不用擔心。** |  |  |  |  |  |  |
| 33A | D：わかりました。まずは検診の手配をお手伝いします。検査は2つあります。1つは24時間心電図、もう1つは心臓超音波検査です。後で両方の用紙をお渡しください。もちろん、もし今朝中に機械の設置が必要であれば、お食事を済ませて、午後に心臓超音波検査を受けに来てください。設置が完了していれば、私も検査できます。ご心配なく。 |  |  |  |  |  |  |
| 33B | D：わかりました。まずは検診の手配をお手伝いします。検査は2つあります。1つは24時間心電図、もう1つは心臓超音波検査です。後で両方の用紙をお渡しください。もちろん、もし今朝中に機械の設置が必要であれば、お食事を済ませて、午後に心臓超音波検査を受けに来てください。設置が完了していれば、私も検査できます。ご心配なく。 |  |  |  |  |  |  |
| 33C | D：はい、ではお伝えしますね。まず検査を2つ手配します。ひとつは「24時間ホルター心電図」、もうひとつは「心臓エコー（超音波検査）」です。このあと、2枚の検査依頼書をお渡ししますので、どちらも提出してください。それから、もしホルター心電図の機器を今朝のうちに装着しないといけないという場合は、いったんご飯を食べに行ってから、午後に心臓エコーを受けに来てください。機器を装着したままでもエコー検査はできますので、心配しなくて大丈夫ですよ。 |  |  |  |  |  |  |
| **34** | **D：（應聲）我跟你說，我先幫你齁，就是做詳細的檢查，包括要留一個24小時的尿液，然後⋯⋯但是這樣子檢查完最快最快啦，大概要⋯⋯兩周再回來比較確定報告出得來。** |  |  |  |  |  |  |
| 34A | D：(応答しながら) では、まずは私がお手伝いします。24時間尿を採取するなど、詳細な検査が必要です。その後…この検査方法が一番早く終わりますが、報告書を確認するためにまた来院されるまで…約2週間かかります。 |  |  |  |  |  |  |
| 34B | D：(応答しながら) では、まずは私がお手伝いします。24時間尿を採取するなど、詳細な検査が必要です。その後…この検査方法が一番早く終わりますが、報告書を確認するためにまた来院されるまで…約2週間かかります。 |  |  |  |  |  |  |
| 34C | D：（うん）じゃあこうしましょう。まずは詳しく検査をしますね。24時間尿も取ってもらいます。それで……この検査を全部終えて、結果がしっかり出るのは、早くても2週間後くらいになると思いますので、その頃にもう一度来てもらえれば確実です。 |  |  |  |  |  |  |
| **35** | **D：那沒問題。那既然你描述的已經很清楚了齁，那，我就幫你排幾個比較重要的檢查。一個叫24小時心電圖，那個抓心率不整。喔它掛在你身上一整天，當天不能洗澡隔天拆掉。那心臟超音波幫你同一個時間一起拍，好不好？啊你什麼時候比較方便？** |  |  |  |  |  |  |
| 35A | D：大丈夫ですよ。とても分かりやすく説明していただいたので、いくつか重要な検査の手配をお手伝いします。一つは24時間心電図検査で、不整脈を検出します。あ、これは一日中装着しているので、当日はシャワーを浴びることはできませんし、翌日には外さなければなりません。それから、心臓の超音波検査も同時に行いますね。それでは、いつがご都合よろしいでしょうか？ |  |  |  |  |  |  |
| 35B | D：大丈夫ですよ。とても分かりやすく説明していただいたので、いくつか重要な検査の手配をお手伝いします。一つは24時間心電図検査で、不整脈を検出します。あ、これは一日中装着しているので、当日はシャワーを浴びることはできませんし、翌日には外さなければなりません。それから、心臓の超音波検査も同時に行いますね。それでは、いつがご都合よろしいでしょうか？ |  |  |  |  |  |  |
| 35C | D：では問題ないですね。もうお話は十分によく分かりましたので、いくつか大事な検査を手配しますね。一つは「24時間ホルター心電図」といって、不整脈をチェックする検査です。これは1日中身体に装着して、当日はシャワーを控えてもらって、翌日に取り外します。そして心臓の超音波（エコー）検査も、そのタイミングで一緒に行いましょうか。ご都合の良い時間帯はいつですか？ |  |  |  |  |  |  |
| **36** | **P：然後這一次吃了，哇，還是很痛。然後我又再吃了⋯⋯嗯⋯⋯止痛藥也還是很痛，然後我就去找甲醫師。然後後來他說，是⋯⋯腸胃炎，然後還給我打針。** |  |  |  |  |  |  |
| 36A | P：今回も飲んでみたんですが、ああ、まだ痛かったんです。それでまた…まあ…鎮痛剤を飲んだんですが、それでも痛かったんです。それでA先生のところに行ったら…胃腸炎だと言われて、注射を打ってくれました。 |  |  |  |  |  |  |
| 36B | P：今回も飲んでみたんですが、ああ、まだ痛かったんです。それでまた…まあ…鎮痛剤を飲んだんですが、それでも痛かったんです。それでA先生のところに行ったら…胃腸炎だと言われて、注射を打ってくれました。 |  |  |  |  |  |  |
| 36C | P：でも今回は飲んでも、うわぁ、まだすごく痛くて。それでまた……うーん、痛み止めも飲んだけど、やっぱり痛くて、それで甲先生のところに行ったんです。そしたら先生が「胃腸炎ですね」って言って、注射もしてくれました。 |  |  |  |  |  |  |
| **37** | **P：對。啊咳不會說很嚴重，就是說這樣「咳咳」，有時候『咳！』這樣。有時候講話就覺得有痰這樣。** |  |  |  |  |  |  |
| 37A | P：はい。ひどい咳ではなく、「咳咳」か、時には「咳！」という程度です。話す時に痰が絡むような感じがする時もあります。 |  |  |  |  |  |  |
| 37B | P：はい。ひどい咳ではなく、「咳咳」か、時には「咳！」という程度です。話す時に痰が絡むような感じがする時もあります。 |  |  |  |  |  |  |
| 37C | P：はい。咳はそんなにひどくはないんですけど、「ゴホゴホ」って感じで、たまに「ゴホッ！」って強く出ることもあります。あと、話していると痰がある感じがすることがあります。 |  |  |  |  |  |  |
| **38** | **D：或是以前⋯⋯留下來的疤啊。就是⋯⋯啊感冒啊，肺炎、發炎留下來的疤都有可能。齁。所以我們目前來說的話，就是會先幫你做，第一個幫你做電腦斷層，第二個幫你抽血，齁，抽一些癌指數啊過敏指數，第三個幫你做肺功能。好不好？** |  |  |  |  |  |  |
| 38A | D：あるいは、風邪、肺炎、炎症などの過去の傷跡かもしれませんね。それでは、まずCTスキャン、それから血液検査（うーん）、がんやアレルギーのレベルを調べて、最後に肺機能検査を受けましょう。よろしいでしょうか？ |  |  |  |  |  |  |
| 38B | D：あるいは、風邪、肺炎、炎症などの過去の傷跡かもしれませんね。それでは、まずCTスキャン、それから血液検査（うーん）、がんやアレルギーのレベルを調べて、最後に肺機能検査を受けましょう。よろしいでしょうか？ |  |  |  |  |  |  |
| 38C | D：あるいは、以前の風邪や肺炎などの炎症が原因でできた「あと（瘢痕）」の可能性もあります。ですので、現時点ではまず以下の検査を行います。第一にCT検査（コンピュータ断層撮影）、第二に採血をして腫瘍マーカーやアレルギーの指標を調べます。第三に肺機能検査を行います。よろしいですか？ |  |  |  |  |  |  |
| **39** | **D：會。因為高山症主要怕的⋯⋯。但是高山症通常麻煩的就是，如果真的很嚴重的時候，你就要趕快下山，然後你那時候可以吸⋯⋯**  **F：我們團進團出，沒辦法。**  **D：可以吸一些藥。對。你去的那個是海拔很高的地方嗎？**  **（請評比底線部份的譯文）** |  |  |  |  |  |  |
| 39A | D：ええ。高山病の一番の問題は…でも、高山病の厄介なところは、本当にひどい場合は、すぐに山を下りないと呼吸ができなくなることです… ／D: 薬を飲んでもいいんですね。そうですね。あなたが行った場所は標高の高いところですか？ |  |  |  |  |  |  |
| 39B | D：ええ。高山病の一番の問題は…でも、高山病の厄介なところは、本当にひどい場合は、すぐに山を下りないと呼吸ができなくなることです… ／D: 薬を飲んでもいいんですね。そうですね。あなたが行った場所は標高の高いところですか？ |  |  |  |  |  |  |
| 39C | D：そうですね。高山病で一番心配なのは、やっぱり重症化した場合なんです。でも本当にひどくなったときは、すぐに下山する必要があります。そのときは吸入薬を使えばいいんです。／D：そういうときは、吸入薬を使えば大丈夫です。ところで、行かれる場所はかなり標高が高いところなんですか？ |  |  |  |  |  |  |
| **40** | **D：它很怕變成潛伏性肺結核。什麼叫潛伏性肺結核？潛伏性肺結核就是說，肺結核菌進到你身體裡面後**  **P：嗯。**  **D：你抵抗力很強**  **P：嗯。**  **D：就把它壓⋯⋯制住**  **（請評比底線部份的譯文）** |  |  |  |  |  |  |
| 40A | D：潜在性結核になる可能性が非常に高いです。潜在性結核とは何ですか？潜在性結核とは、結核菌が体内に侵入した後  D：抵抗力が強いですね  D：抑えるだけだよ… |  |  |  |  |  |  |
| 40B | D：潜在性結核になる可能性が非常に高いです。潜在性結核とは何ですか？潜在性結核とは、結核菌が体内に侵入した後  D：抵抗力が強いですね  D：抑えるだけだよ… |  |  |  |  |  |  |
| 40C | D：それが潜伏性結核になるのを心配する人もいます。潜伏性結核っていうのは何かというと、結核菌が体の中に入ったあと、  D：あなたの免疫力が強ければ、  D：菌を抑え込んで、発症しないようにすることができます。 |  |  |  |  |  |  |

Physicians are denoted by “D,” patients by “P,” and accompanying family members by “F.”

Please rate translation accuracy on a 6-point scale with end points ranging from 0 to 5, where 5 indicates the highest level of accuracy. Because medical interpreters may prioritize different aspects of accuracy, please base your rating on your own professional judgment.

All source materials are derived from authentic physician-patient interactions and are presented in their original spoken Chinese form. Because real-world clinical conversations may include incomplete or ambiguous expressions, no linguistic modifications were made to the transcripts. This approach allows you to evaluate artificial intelligence–generated translations under realistic clinical communication conditions.
