# Supplementary material for: Comparative Evaluation of ChatGPT, Google Translate, and UD Talk for Chinese-to-Japanese Translation in Cardiology and Pulmonology Outpatient Consultations: Prospective Observational Study
Source: J Med Internet Res. 2026 Jun 18;28:e93082. doi: 10.2196/93082 (PMC13329329; doi:10.2196/93082)
Supplement: Multimedia Appendix 2 [file jmir_v28i1e93082_app2.docx]

**Multimedia Appendix 2.** Instrument for evaluating the lay participant–rated satisfaction with the translations.

| **No.** | **Content** | **Rating** | | | | | |
| --- | --- | --- | --- | --- | --- | --- | --- |
|  |  | **0** | **1** | **2** | **3** | **4** | **5** |
| **1** | **D：嘿。哈囉，最近還好嗎？你那個藥吃的怎麼樣？人有沒有不舒服？** |  |  |  |  |  |  |
| 1A | D：やあ。こんにちは。最近調子はどうですか？薬はどうですか？体調が悪い人はいませんか？ |  |  |  |  |  |  |
| 1B | D：やあ、こんにちは。調子はどう？薬は大丈夫？体調は悪い？ |  |  |  |  |  |  |
| 1C | D：やあ、こんにちは。最近はどうですか？あのお薬はちゃんと飲めていますか？体調に変わりはありませんか？ |  |  |  |  |  |  |
| **2** | **P：他說是發作再吃** |  |  |  |  |  |  |
| 2A | P：襲われた時に食べたと言ってました。 |  |  |  |  |  |  |
| 2B | P：攻撃が起こったらそれを取ると言っていました。 |  |  |  |  |  |  |
| 2C | P：発作が起きた時に飲むようにと言われました。 |  |  |  |  |  |  |
| **3** | **F：他的指、腎指數真的是非常不好，真的不能再打針了。** |  |  |  |  |  |  |
| 3A | F：彼の指と腎臓の人差し指の状態は本当に悪く、もう注射を受けることができません。 |  |  |  |  |  |  |
| 3B | F：彼の指と腎臓の人差し指の状態は本当に悪く、もう注射を受けることは本当に不可能なんです。 |  |  |  |  |  |  |
| 3C | F：彼の腎機能の数値は本当に悪くて、もう注射は絶対にダメだって言われたんです。 |  |  |  |  |  |  |
| **4** | **P：現在我就擔心這個頭一直脹脹的，不知道什麼⋯⋯** |  |  |  |  |  |  |
| 4A | P：頭がずっと腫れているのが心配なんです。どうしてなのかわからないんですけど… |  |  |  |  |  |  |
| 4B | P：頭が腫れている理由が心配です。何が起こっているのか分かりません… |  |  |  |  |  |  |
| 4C | P：今はこの頭がずっとぼーっとする感じが気になっていて……何が原因なのか分からなくて。 |  |  |  |  |  |  |
| **5** | **D：（應聲）妳有沒有回過門診？去看過？***  **情境說明：醫生詢問病人在之前的診所看病時，有沒有回診看過心臟問題。** |  |  |  |  |  |  |
| 5A | D：(答える) クリニックに戻ったことはありますか？行ったことはありますか？ |  |  |  |  |  |  |
| 5B | D：(応答) クリニックに戻ったことはありますか? クリニックに行ったことはありますか? |  |  |  |  |  |  |
| 5C | D：（うなずきながら）外来には戻って診てもらったことがありますか？通院されましたか？ |  |  |  |  |  |  |
| **6** | **D：（應聲）妳有沒有回過門診？去看過？ P：我，有啊。我就一直在看哪。**  **（請評比底線部份的譯文）** |  |  |  |  |  |  |
| 6A | P：はい、私もずっと見ていました。 |  |  |  |  |  |  |
| 6B | P：ええ、私も。ただ見ていたんです。 |  |  |  |  |  |  |
| 6C | P：はい、あります。ずっと通院していますよ。 |  |  |  |  |  |  |
| **7** | **D：那妳有做過心臟，他有給妳做過心臟超音波嗎？***  **（情境說明：醫生詢問病人在其它診所時，有沒有做過心臟超音波檢查）** |  |  |  |  |  |  |
| 7A | D：心臓手術を受けたことはありますか？心臓の超音波検査は受けましたか？ |  |  |  |  |  |  |
| 7B | D：心臓スキャンを受けたことはありますか？超音波検査も受けましたか？ |  |  |  |  |  |  |
| 7C | D：それで、心臓のエコー検査（心臓超音波）は、受けたことがありますか？先生がしてくれましたか？ |  |  |  |  |  |  |
| **8** | **D：沒有，現在健保⋯⋯其實換醫生齁，還是建議自己要帶資料過來。**  **P：嗯。**  **D：因為那樣子才不會搞錯。健保資料很陽春，常常又當機連不上去。**  **（請評比底線部份的譯文）** |  |  |  |  |  |  |
| 8A | D：そうすれば間違いがなくなるからです。健康保険の情報は非常に基本的なもので、よくクラッシュして接続できないんです。 |  |  |  |  |  |  |
| 8B | D：そうすれば間違いが起こらないからです。健康保険の情報は非常に基本的なもので、頻繁にクラッシュしたり接続できなかったりします。 |  |  |  |  |  |  |
| 8C | D：そうすれば間違いも防げますからね。健康保険のデータはけっこう簡易的で、よく接続できなくなったりもしますし。 |  |  |  |  |  |  |
| **9** | **D：啊甲醫師有說要追蹤嗎？** |  |  |  |  |  |  |
| 9A | D：A医師は経過観察が必要だと言いましたか？ |  |  |  |  |  |  |
| 9B | D：A医師は経過観察したいと言っていましたか？ |  |  |  |  |  |  |
| 9C | D：ああ、甲先生はフォローアップ（経過観察）するように言ってましたか？ |  |  |  |  |  |  |
| **10** | **P：胃、胃在晚一點的時候，還是我喝茶喝咖啡的原因？**  **D：你說會緊緊的嗎？**  **（請評比底線部份的譯文** |  |  |  |  |  |  |
| 10A | P：胃、一日の終わりに胃が痛くなる、それともお茶やコーヒーを飲むからでしょうか? |  |  |  |  |  |  |
| 10B | P：それは胃のせいでしょうか、それとも夜遅くに飲むお茶やコーヒーのせいでしょうか? |  |  |  |  |  |  |
| 10C | P：胃が…遅い時間になるとちょっと…。それとも、お茶とかコーヒーを飲むせいですか？ |  |  |  |  |  |  |
| **11** | **D：好了。我跟你說，我這一次齁，也不要開太久啦，因為你胃還在不舒服，而且有一些檢查也還沒做完整。我們一個月回來好不好？**  **（情境說明：醫師開藥）** |  |  |  |  |  |  |
| 11A | D：分かりました。実は、お腹がまだ痛くて、検査もまだ終わっていないので、今回はあまり長く開けたままにしないようにしています。1ヶ月後にまた来ていただけますか？ |  |  |  |  |  |  |
| 11B | D：分かりました。実は、今回はあまり長く空けておくつもりはありません。お腹がまだ痛くて、検査もまだ終わっていないので。1ヶ月後にまた来ていただけますか？ |  |  |  |  |  |  |
| 11C | D：じゃあ今回は、あまり間隔を空けずにしておきましょう。胃の調子もまだ良くないし、いくつかの検査もまだ終わってないですからね。1ヶ月後にもう一度来てもらうということで、どうですか？ |  |  |  |  |  |  |
| **12** | **D：齁。好。那就這樣子囉。齁，那我們就先檢查完整，啊你也測一下血壓。下次把藥物帶過來，那我們確定一下，看⋯到時候的計劃怎麼定，這樣好不好？** |  |  |  |  |  |  |
| 12A | D：ああ。分かりました。それで終わりです。あ、まずは精密検査をしましょう。血圧も測れますよ。次回薬を持ってきてください。それで確認して…どう計画を立てるか…それでいいですか？ |  |  |  |  |  |  |
| 12B | D：ああ。分かりました。それで終わりです。あ、まずは精密検査をしましょう。血圧も測れますよ。次回薬を持ってきてください。それで確認して、どうするか決めましょう。よろしいですか |  |  |  |  |  |  |
| 12C | D：うん、じゃあ今日はこれで終わりにしましょう。まずはしっかり検査をして、あなたも血圧を測ってください。次回来るときにはお薬も持ってきてくださいね。それで、今後の治療方針を一緒に確認しましょう。いいですか？ |  |  |  |  |  |  |
| **13** | **D：胃藥**  **P：應該可以不開了。**  **D：可以不用了啦齁。好**  **（請評比底線部份的譯文）** |  |  |  |  |  |  |
| 13A | D：大丈夫ですよ。 |  |  |  |  |  |  |
| 13B | D：大丈夫だよ。OK。 |  |  |  |  |  |  |
| 13C | D：もう必要ないですね。はい。 |  |  |  |  |  |  |
| **14** | **D：我們大概就是一兩年齁，就讓你再穩定一兩年。那個跑步心電圖跟心臟超音波都要再追蹤。你跑步...有辦法跑步啦齁？** |  |  |  |  |  |  |
| 14A | D：おそらく1、2年待って、その後さらに1、2年かけて状態を安定させます。心電図と心臓超音波検査を再度記録します。...逃げる方法はあるんですか？ |  |  |  |  |  |  |
| 14B | D：停滞期間はおそらく1、2年程度でしょうから、あと1、2年は安定期に入るまで待ちましょう。ランニング心電図と心臓超音波検査を再度記録します。走ってください。...逃げる方法はあるんですか？ |  |  |  |  |  |  |
| 14C | D：だいたい1〜2年ごとに、安定していれば心電図（運動負荷付き）と心臓エコーの両方を再検査します。運動は…...走ったりするのは大丈夫そうですか？ |  |  |  |  |  |  |
| **15** | **D：妳那樣發作過幾次？ P：很多次。 D：很多次喔。 P：只是最⋯⋯，這是比較最近的，的這兩次的，就是3月16**  **（請評比底線部份的譯文）** |  |  |  |  |  |  |
| 15A | P：ただ、一番最近の…これが一番最近の 2 つのもので、3 月 16 日のものでした。 |  |  |  |  |  |  |
| 15B | P：でも、一番最近の 2 回は 3 月 16 日でした。 |  |  |  |  |  |  |
| 15C | P：ただ、最近のは特にこの2回で……3月16日です。 |  |  |  |  |  |  |
| **16** | **D：哦哦。現在比較沒有咳？**  **P：現在⋯⋯咳嗽，沒有。**  **（請評比底線部份的譯文）** |  |  |  |  |  |  |
| 16A | D：ああ、咳は治まりましたか？ |  |  |  |  |  |  |
| 16B | D：あらまあ。咳は治まりましたか？ |  |  |  |  |  |  |
| 16C | D：ああ、今はあまり咳は出ていませんか？ |  |  |  |  |  |  |
| **17** | **D：因為有一些，誒⋯⋯有一些藥我們還是要等確切檢查結果出來了，再開比較好。** |  |  |  |  |  |  |
| 17A | D：ええと、処方する前に正確な試験結果が出るまで待たなければならない薬がいくつかあるからです。 |  |  |  |  |  |  |
| 17B | D：ええと、いくつかの薬は、処方する前に正確な検査結果が出るまで待たなければなりません。 |  |  |  |  |  |  |
| 17C | D：というのも、いくつかのお薬については、やはり検査結果がはっきり出てから処方した方がいいので。 |  |  |  |  |  |  |
| **18** | **D：這樣好了啦，我開一個緊急的吸劑給你。緊急的就是說如果你真的比較喘的時候可以吸。藍色的，你有沒有拿過？** |  |  |  |  |  |  |
| 18A | D：わかりました。緊急用吸入器を処方します。緊急用というのは、本当に息切れがひどい時に使うものです。青い色の吸入器ですね。使ったことはありますか？ |  |  |  |  |  |  |
| 18B | D：わかりました。緊急用の吸入器を処方します。息切れがひどい時に使うものです。青い色のものです。使ったことはありますか？ |  |  |  |  |  |  |
| 18C | D：ではこうしましょう。緊急用の吸入薬をひとつ出しておきますね。本当に息苦しくなったときに使えるものです。青い吸入薬なんですが、使ったことありますか？ |  |  |  |  |  |  |
| **19** | **D：（安排檢查）看看妳有沒有氣管過敏啊等等，**  **P：嗯。**  **D：好不好？齁。那妳現在就要吃藥嗎？還是等我們檢查出來了，確定什麼問題再來吃藥？**  **（請評比底線部份的譯文）** |  |  |  |  |  |  |
| 19A | D：わかりましたか？痛いですね。今すぐ薬を飲まないといけないですか？それとも、症状が確認できるまで待った方がいいですか？ |  |  |  |  |  |  |
| 19B | D：わかりましたか？痛いですね。今すぐ薬を飲まないといけないですか？それとも、検査して何が問題なのかわかるまで待った方がいいですか？ |  |  |  |  |  |  |
| 19C | D：いいですか？じゃあ今すぐお薬を飲みたいですか？それとも検査して原因がはっきりしてから薬を使いたいですか？ |  |  |  |  |  |  |
| **20** | **P：那檢查是現在檢查還是？** |  |  |  |  |  |  |
| 20A | P：今検査を受けたほうがいいですか? |  |  |  |  |  |  |
| 20B | P：今チェックを受け取ってもいいですか? |  |  |  |  |  |  |
| 20C | P：検査は今すぐ行うんですか？それとも……？ |  |  |  |  |  |  |
| **21** | **D：那妳是做X光還是低輻射電腦斷層？**  **P：低輻射。**  **D：做低輻射啦。**  **P：對。**  **D：我幫妳看一下。**  **P：然後我在兩年前有在臺安做。但，臺安那時候是說⋯⋯。在臺安做⋯⋯**  **（請評比底線部份的譯文）** |  |  |  |  |  |  |
| 21A | P：それで2年前に泰安でやったんですよ。でもその時、泰安は…って言ったんですよ |  |  |  |  |  |  |
| 21B | P：それから2年前に泰安でやりました。でもその時、泰安は…泰安で働いてるって…って言ってたんです。 |  |  |  |  |  |  |
| 21C | P：それから、2年前にタイアン病院で受けたことがあります。でも、そのときタイアンでは…タイアンで検査を受けたときは… |  |  |  |  |  |  |
| **22** | **F：喔～。沒有，因為那邊寫，你寫6小⋯⋯，那藥袋的明細是寫，6小時吃一次是2顆。** |  |  |  |  |  |  |
| 22A | F：ああ。いや、あそこに書いてあるから、6時間って書いてあるよね…薬袋には6時間ごとに2錠ずつ服用って書いてある。 |  |  |  |  |  |  |
| 22B | F：ああ。いや、あそこに書いてあるから、6時間って書いてあるじゃないですか。薬袋には6時間ごとに2錠ずつ服用するように書いてあるんですよ。 |  |  |  |  |  |  |
| 22C | F：ああ〜。でも、そっちに書いてあるのは、6時間ごとに2錠って書いてあるんですよ。 |  |  |  |  |  |  |
| **23** | **D：然後我們三個月拿一次藥，妳還是要吸藥啦***  **情境說明：醫師指示病人使用吸入型藥物。** |  |  |  |  |  |  |
| 23A | D：それから、3か月に1回薬を服用することになりますが、その薬は引き続き服用しなければなりません。 |  |  |  |  |  |  |
| 23B | D：それから、3か月に1回薬を服用することになりますが、引き続き薬を服用しなければなりません。 |  |  |  |  |  |  |
| 23C | D：それから、お薬は3ヶ月ごとにもらって、吸入はちゃんと続けてくださいね。 |  |  |  |  |  |  |
| **24** | **D：都沒有齁？那妳最近有感冒嗎？**  **P：誒⋯⋯沒有餒。**  **D：都沒有？**  **（請評比底線部份的譯文）** |  |  |  |  |  |  |
| 24A | D：どれもないんですか？ |  |  |  |  |  |  |
| 24B | D：無しですか？ |  |  |  |  |  |  |
| 24C | D：全くなかったんですね？ |  |  |  |  |  |  |
| **25** | **D：嗯～。妳這個禮拜五有時間來做檢查嗎？還是下禮拜？** |  |  |  |  |  |  |
| 25A | D：うーん。今週の金曜日か来週の健康診断に来られる時間はありますか？ |  |  |  |  |  |  |
| 25B | D：うーん。今週の金曜日、それとも来週の検診は受けられますか？ |  |  |  |  |  |  |
| 25C | D：うーん…。今週の金曜日に検査に来る時間はありますか？それとも来週のほうがいいですか？ |  |  |  |  |  |  |

Physicians are denoted by “D,” patients by “P,” and accompanying family members by “F.”

Please rate your overall satisfaction with the translated content on a 6-point scale with end points ranging from 0 to 5, where 5 indicates the highest level of satisfaction.

All source materials are derived from authentic physician-patient interactions and are presented in their original spoken Chinese form. Because real-world clinical conversations may include incomplete or ambiguous expressions, no linguistic modifications were made to the transcripts. This approach allows you to evaluate artificial intelligence–generated translations under realistic clinical communication conditions.
