# Supplementary material for: Comparative Evaluation of ChatGPT, Google Translate, and UD Talk for Chinese-to-Japanese Translation in Cardiology and Pulmonology Outpatient Consultations: Prospective Observational Study
Source: J Med Internet Res. 2026 Jun 18;28:e93082. doi: 10.2196/93082 (PMC13329329; doi:10.2196/93082)
Supplement: Multimedia Appendix 3 [file jmir_v28i1e93082_app3.docx]

**Multimedia Appendix 3.** Subgroup analyses of translation accuracy and satisfaction across translation systems by outpatient specialty.

|  | ChatGPT (0-5), median (IQR) | |  | Google Translate (0-5), median (IQR) | |  | UD Talk (0-5), median (IQR) | |
| --- | --- | --- | --- | --- | --- | --- | --- | --- |
|  | Cardiology | Pulmonology |  | Cardiology | Pulmonology |  | Cardiology | Pulmonology |
| **Accuracy** | 5.0 (4.0-5.0) | 5.0 (4.0-5.0) |  | 2.0 (0.0-3.0) | 3.0 (1.0-4.0)^***^ |  | 2.0 (0.0-3.0) | 2.0 (1.0-4.0)^***^ |
| **Satisfaction** | 5.0 (4.0-5.0) | 5.0 (4.0-5.0) |  | 2.0 (1.0-3.0) | 3.0 (1.0-4.0)^**^ |  | 2.0 (1.0-3.0) | 2.0 (2.0-4.0)^***^ |

Data are presented as median (interquartile range) values.

Comparisons between cardiology and pulmonology outpatient clinics were performed using the Wilcoxon rank-sum test.

^**^*P*<.01 and ^***^*P*<.001.
